# Reoperative CABG in a patient with prior concomitant lung transplantation and two-vessel CABG

**DOI:** 10.21542/gcsp.2023.25

**Published:** 2023-09-30

**Authors:** Emily L. Larson, Anson Y. Lee, Jennifer S. Lawton, Hamza Aziz

**Affiliations:** 1 Division of Cardiac Surgery, Department of Surgery, Johns Hopkins Hospital, 1800 Orleans St, Baltimore, MD 21287, USA; 2 Department of Medicine, John A. Burns School of Medicine, University of Hawai’i, Honolulu, HI, USA

## Abstract

Background: Lung transplants (LTx) are being offered to increasingly older patients, and as a result, more concomitant coronary artery disease is being encountered in LTx candidates. While concurrent coronary artery bypass grafting (CABG) and LTx have become more common, the long-term considerations of reoperative CABG in patients following CABG with concomitant LTx are not fully understood.

Case presentation: A 75-year-old man with a history of bilateral LTx and concomitant CABG X 2 15 years prior presented to the emergency room with tachycardia and chest discomfort radiating to the left upper extremity. Emergent coronary angiography revealed severe three-vessel coronary artery disease with two occluded saphenous vein grafts, severe distal obtuse marginal (OM) and left circumflex disease, a collateralized chronic total occlusion of the mid LAD, and tortuosity of the proximal right innominate artery. The patient underwent a complex redo sternotomy and CABG X 2 due to dense adhesions in the mediastinum and pleura bilaterally. The postoperative course was complicated by left leg SVG harvest site cellulitis treated with IV antibiotics and hypervolemia treated with diuresis. The patient was discharged postoperatively on day 13.

Discussion: To our knowledge, this is the first reported successful reoperative CABG in a patient with a history of concomitant LTx and CABG. This case demonstrates feasibility, though additional caution is required due to the technical complexity and risk of immunosuppression in such complex patients.

## Introduction

Lung transplants (LTx) are an important tool for patients with limited survival due to end-stage lung conditions. Over the past 10 years, the increase in LTx demand has far exceeded that of any other organ transplant^1,2^. With further advances in medical care, LTx are being offered to increasingly older patients, and as a result, more cases of concomitant coronary artery disease (CAD) have also been encountered in LTx candidates^3,4^. It is estimated that more than half of all patients with advanced lung disease also have occult CAD, and with the rising age of LTx recipients, this proportion will likely increase ^5^.

Historically, coronary artery bypass grafting (CABG), a treatment for CAD, was seen as a contraindication to LTx^6–8^. Recently, CABG has been relegated to being a relative contraindication with a range of revascularization options available for LTx patients, such as; percutaneous coronary intervention (PCI), CABG prior to LTx, or concomitant CABG during the transplant ^9^. A number of studies have shown that LTx patients with concurrent CABG procedures had no statistical difference in post-transplant survival compared to LTx patients without CABG^9–12^. Also, 5-year survival rates were comparable in patients who received LTx as well as other concomitant cardiac procedures such as atrial septal defect surgeries ^13^. However, even with comparable mortality rates, concurrent CABG was associated with longer postoperative length of stay, increased time in the intensive care unit, and more postoperative days on ventilator support ^3^.

Moreover, approximately 10–20% of all patients with a prior CABG will need another revascularization procedure within a decade of their initial surgery, due to progression of native disease or vein graft disease ^14^. While previous work has identified increased surgical complexity and operative mortality in redo CABG patients compared to first-time CABG recipients^15–17^, there has not been a study that explored redo CABG in patients following concomitant LTx and CABG. Therefore, we present a report of a successful reoperative CABG in a patient with previous concurrent two-vessel CABG and bilateral lung transplant.

## Case report

The patient is a 75-year-old man with a history of bilateral LTx and concomitant CABG 15 years prior to presentation who underwent a redo CABG. In the first procedure, a two-vessel CABG with saphenous veins to the left anterior descending (LAD) and LAD diagonal arteries was performed at the time of bilateral LTx for bronchiectasis. The patient’s immunosuppressive regimen included prednisone, tacrolimus, and azathioprine.

One year later, the patient underwent percutaneous intervention for a saphenous vein graft (SVG) occlusion with a drug-eluting stent. The patient’s medical history also includes hypertension, stage II chronic kidney disease, several opportunistic pneumonias following LTx, resolved COVID-19 infection two months prior to presentation, and deep vein thrombosis.

**Figure 1.**
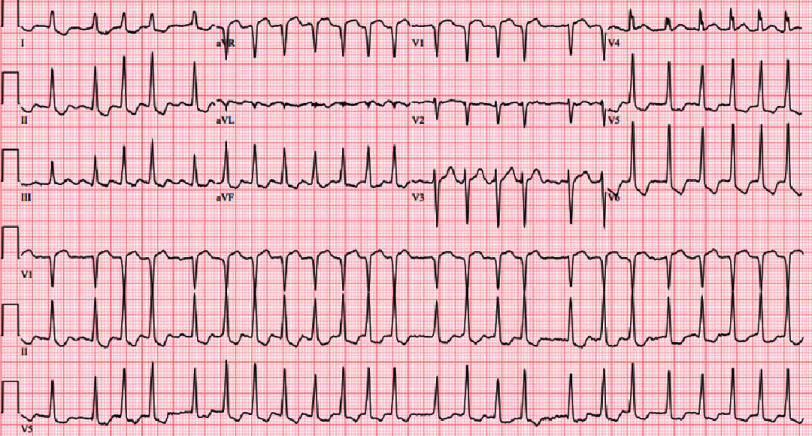
ECG at presentation for a patient with prior concomitant CABG and bilateral lung transplant. Atrial fibrillation with rapid ventricular response. Diffuse ST depressions, and ST elevation in lead aVR. Abbreviations: Electrocardiogram (ECG), Coronary artery bypass graft (CABG).

The patient presented to the emergency room with tachycardia and chest discomfort radiating to the left upper extremity. The initial electrocardiogram (ECG) showed atrial fibrillation with rapid ventricular response and diffuse ST depressions and ST elevation in lead aVR (Figure 1). A subsequent ECG, with ongoing chest discomfort, showed sinus rhythm with submillimeter ST elevations in leads III and aVR and lateral ST depressions and T-wave inversions.

**Figure 2.**
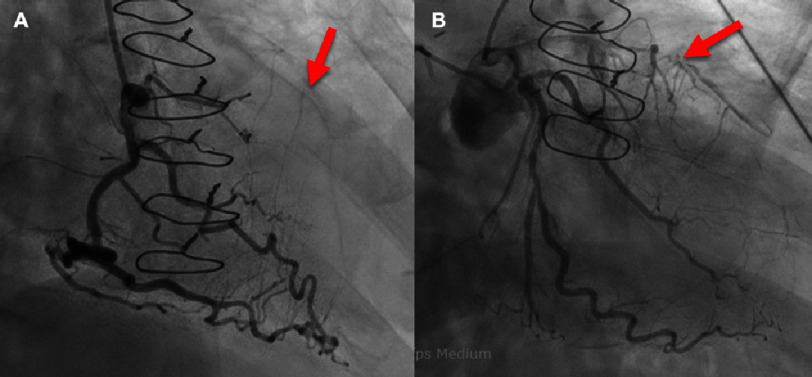
Preoperative coronary angiography of a patient with prior concomitant CABG and bilateral lung transplant presenting for a redo CABG. Sternal wires noted. (A) RCA with no significant stenosis, right dominant system, and faint collateralization to the LAD (red arrow). (B) Proximal LAD with 90% stenosis and total occlusion in the mid segment (red arrow). Proximal LCX has 70 to 80% stenosis, OM1 has ostial eccentric 80 to 90% stenosis; OM2 has mid segment 80 to 90% stenosis. Previous vein grafts occluded (injections not shown). Abbreviations: Coronary artery bypass graft (CABG), Right coronary artery (RCA), Left anterior descending (LAD), Left circumflex artery (LCX); Obtuse marginal (OM).

**Figure 3.**
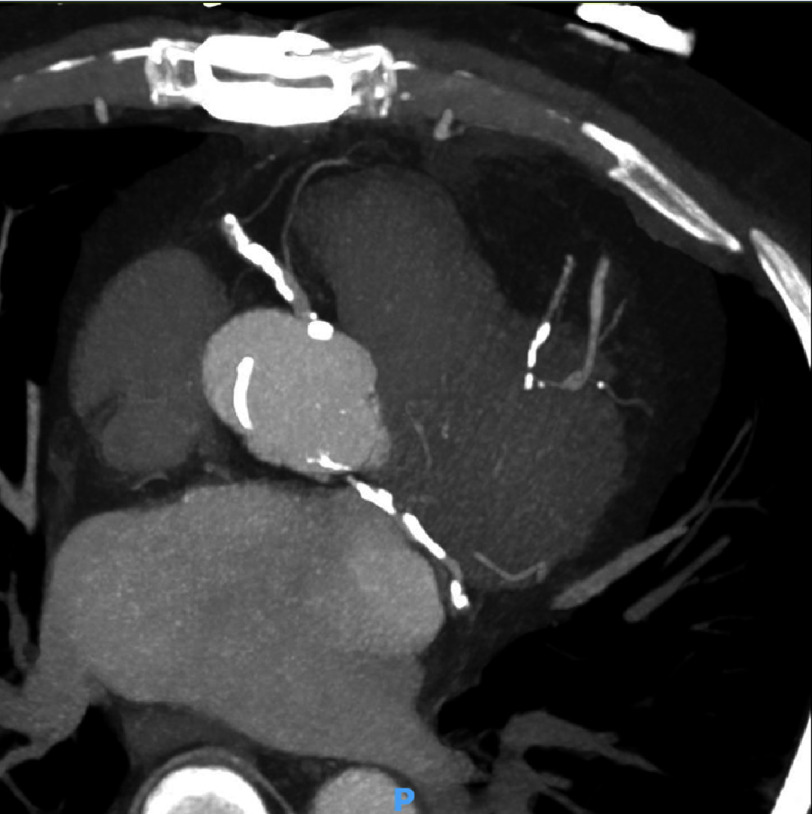
Preoperative axial CTA of heart in patient with prior concomitant CABG and bilateral lung transplant presenting for redo CABG. Calcification of aorta, RCA, and LCA. Both saphenous vein grafts occluded. Abbreviations: CT angiography (CTA), Coronary artery bypass graft (CABG), Right coronary artery (RCA), Left coronary artery (RCA).

He was administered aspirin, heparin, and ticagrelor and then underwent emergent coronary angiography (Figure 2). The study revealed severe three-vessel coronary artery disease with occluded saphenous vein grafts (aorta to LAD and aorta to diagonal), severe distal obtuse marginal (OM) and left circumflex disease, a collateralized chronic total occlusion of the mid LAD. Preoperative CT angiography showed calcification of the aorta and both coronary arteries as well as occlusion of both vein grafts (Figure 3). Transthoracic echocardiogram demonstrated mildly depressed ejection fraction of 50%. Pulmonary function tests showed FEV1 of 2.98 L (102% predicted) and DLCO of 13.08 mL/min/mmHg (57% predicted).

The patient underwent a redo sternotomy. There were dense adhesions in the mediastinum and pleura bilaterally. The aorta and the right atrium were cannulated. The prior saphenous vein graft to the obtuse marginal artery was transected to be able to apply the cross clamp. Otherwise, the previous vein grafts were not manipulated to avoid debris embolizing into the coronaries. Two vessel CABG with the *in situ* left internal mammary artery (LIMA) anastomosed distal to the prior vein graft on the LAD. Saphenous vein graft to the obtuse marginal artery was anastomosed proximally on the ascending aorta. Post-bypass function was preserved.

The postoperative course was complicated by left leg SVG harvest site cellulitis treated with IV antibiotics and hypervolemia treated with diuresis. The patient was discharged home on postoperative day 13. The patient had no further complications post-discharge. At one year follow-up, the patient was doing well, with no reported symptoms of angina or decompensated heart failure and was well-perfused and euvolemic on physical exam.

## Discussion

As LTx criteria broaden to include older patients, clinically significant CAD is encountered with increasing frequency in LTx patients^3,4^. Although significant CAD requiring CABG was historically a contraindication to LTx, it is now only a relative contraindication^6–9^. Concomitant LTx and cardiac procedures have no associated increased mortality compared to LTx alone though are associated with increased length of stay^9–13^. As patients live longer after concomitant LTx and CABG, they can be expected to require redo coronary revascularization that is associated with increased operative complexity and mortality^14–17^. The patient described had dense adhesions throughout the chest, which increased the technical difficulty of the procedure.

In addition to the isolated risk posed by redo CABG, previous CABG-LTx patients also face the additional risk associated with long term immunosuppression. The patient reported was on a maintenance steroid, a calcineurin inhibitor, and a nucleotide blocking agent, which may have contributed to the postoperative SVG harvest site infection that complicated his postoperative course and extended his length of stay.

## What have we learned?

To our knowledge, this case characterizes the first reported successful redo CABG in a patient with previous concomitant LTx and CABG. The reported case demonstrates feasibility of coronary revascularization in these complex, high-risk patients.
